# Surveillance of wild animals carrying infectious agents based on high-throughput screening platform in the Republic of Korea

**DOI:** 10.1186/s12917-023-03714-0

**Published:** 2023-09-14

**Authors:** Myeongsu Kim, Jieun Bae, Byungkwan Oh, Haerin Rhim, Myeon-Sik Yang, Somyeong Yang, Bumseok Kim, Jae-Ik Han

**Affiliations:** 1 Laboratory of Wildlife Medicine, College of Veterinary Medicine, Jeonbuk National University, Iksan, 54596 Republic of Korea; 2 Laboratory of Veterinary Pathology, College of Veterinary Medicine, Jeonbuk National University, Iksan, 54596 Republic of Korea; 3 Jeonbuk Wildlife Center, Jeonbuk National University, Iksan, 54596 Republic of Korea

**Keywords:** High-throughput platform, Infectious disease, Reservoir, Wild animal

## Abstract

**Background:**

Infectious diseases transmitted by wild animals are major threats to public health. This study aimed to investigate the potential of rescued wild animals that died of unknown causes as reservoirs of infectious agents. From 2018 to 2019, 121 dead wild animals (55 birds and 66 mammals) were included in this study. All wild animals died during treatment after anthropogenic events. After deaths of animals, necropsies were performed and trachea, lungs, large intestine (including stool), and spleen were collected to determine causes of deaths. A high-throughput screening (HTS) quantitative polymerase chain reaction (qPCR) designed to detect 19 pathogens simultaneously against 48 samples in duplicate was performed using nucleic acids extracted from pooled tissues and peripheral blood samples. If positive, singleplex real-time PCR was performed for individual organs or blood samples.

**Results:**

The HTS qPCR showed positive results for *Campylobacter jejuni* (10/121, 8.3%), *Campylobacter coli* (1/121, 0.8%), *Mycoplasma* spp. (78/121, 64.5%), and *Plasmodium* spp. (7/121, 5.7%). Singleplex real-time PCR confirmed that *C. jejuni* was detected in the large intestine but not in the blood. *C. coli* was only detected in the large intestine. *Mycoplasma* spp. were detected in all organs, having the highest proportion in the large intestine and lowest in the blood. *Plasmodium* spp. was also detected in all organs, with proportions being were similar among organs.

**Conclusions:**

This study shows that wild animals can become carriers of infectious agents without showing any clinical symptoms.

**Supplementary Information:**

The online version contains supplementary material available at 10.1186/s12917-023-03714-0.

## Background

More than half of known infectious pathogens are zoonotic, 12% of which are causing emerging infectious diseases (EIDs) [1]. The number and diversity of diseases are increasing. Many diseases not only threaten human health, but also affect livestock and wild animals [2]. Globalization, environmental change, and migrating birds or wild animals living in borders can spread diseases or pathogens more easily and widely. For example, avian influenza (AI) is one of the most well-known viral diseases transmitted from migratory birds to poultry [3]. Climate change increases the risk of new inflows of wildlife-derived infectious diseases, especially arthropod-borne diseases such as malaria and Japanese encephalitis. It also increases the spread of disease [4]. The trade of wild animals and wild animal products can also affect the spread of diseases [5].

Most EIDs are considered to spread from wildlife. The importance of constructing surveillance programs and research is increasing [6]. Outbreaks of infectious diseases are recognized mainly by reporting them in clinical cases or by detecting pathogens extracted from the environment or carcasses. Constructing a surveillance program to enable rapid detection and response can help prevent the spread of diseases [7, 8]. High-throughput quantitative polymerase chain reaction (qPCR) platforms are useful because large-scale detection can be performed rapidly and sensitively [9, 10].

The importance of surveilling wildlife-derived infectious diseases is increasing, as is the number of diseases that require surveillance. Monitoring all diseases takes substantial time, finances, and reagent volume. Moreover, research on untamed and living wild animals requires greater effort [11]. Although the gold standard varies from disease to disease, molecular diagnosis is commonly considered a useful method [12]. Real-time qPCR is often used to detect pathogens because it is capable of performing quantitative analysis with a high-sensitivity [13]. However, with increasing number of diseases requiring surveillance, it is costly and time-consuming to test all samples for significant results [14]. A high-throughput qPCR platform can be used to address this issue to reduce time and cost. Its analytical sensitivity and specificity are not decreased compared to those of singleplex screening [9].

This study was conducted to investigate the potential of wild animals rescued from traumatic distress and died during treatment or were euthanized as carriers of infectious diseases using a high-throughput qPCR platform.

## Results

### Results of high-throughput qPCR platform

Results of high-throughput qPCR platform showed that samples were positive for *Campylobacter jejuni* (10/121, 8.3%), *Campylobacter coli* (1/121, 0.8%), *Mycoplasma* spp. (78/121, 64.5%), and *Plasmodium* spp. (7/121, 5.7%) (Table 1). *C. coli* was detected only in a kestrel. *C. jejuni* and *C. coli* were detected in water birds or other wild animals that had been rescued around water bodies such as rivers and seas. *Mycoplasma* spp. and *Plasmodium* spp. were detected in various species from rescued areas such as expressways, residential areas, and farmlands.

**Table 1:** Prevalence of pathogen and reason for rescue of animals with pathogens detected

Pathogen		No. (%)	Species	No. of animal	Reason for rescue
*Camphylobacter jejuni*	Birds	8 (14.5)	Green-backed heron	1	Hit by car
			Cattle egret	4	Collision (3) Hit by car (1)
			Sparrow hawk	1	Collision
			Jay	1	Missing child
			Goshawk	1	Collision
	Mammals	2 (3)	Korean water deer	1	Hit by car
			Raccoon dog	1	Hit by car
*Camphylobacter coli*	Birds	1 (1.8)	Kestrel	1	Collision
*Mycoplasma* spp.	Birds	38 (69.1)	Green-backed heron	1	Hit by car
			Chinese little bittern	1	Hit by car
			Oriental turtle dove	1	Collision
			Hummingbird	1	Unknown
			Sparrow hawk	2	Hunting (1) Collision (1)
			Scops owl	1	Collision
			Brown hawk-owl	4	Collision (4),
			Eagle owl	4	Hit by car (1) Collision (1) Missing child (1) Unknown (1)
			Jay	1	Missing child (1)
			Gray heron	5	Missing child (1) Collision (1) Starvation and exhaustion (2) Unknown (1)
			Large egret	1	Collision (1)
			Intermediate egret	1	Missing child (1)
			Goshawk	3	Hit by car (1) Collision (1) Hunting (1)
			Cattle egret	6	Collision (5) Unknown (1)
			Hoopoe	1	Collision
			Kestrel	4	Collison (2) Hit by car (1) Unknown (1)
			Spot-billed duck	1	Missing child
	Mammals	40 (60.6)	Korean water deer	23	Hit by car (18) Missing child (2) Hunting (1) Starvation and exhaustion (1) Unknown (1)
			Raccoon dog	17	Hit by car (4) Infection (8) Hunting (1) Biting (2) Unknown (2)
*Plasmodium* spp.	Birds	6 (10.9)	Chinese Little Bittern	1	Hit by car
			Spot-billed duck	1	Missing child
			Brown hawk-owl	1	Collision
			Jay	1	Missing child
			Cattle egret	1	Collision
			Eurasian woodcock	1	Collision
	Mammals	1 (1.5)	Korean water deer	1	Hit by car

### Results of singleplex qPCR

In birds, singleplex qPCR revealed that *C. jejuni* was detected in 100% of large intestine samples, 63% of trachea samples, 75% of lung samples, and 50% of spleen samples. *C. coli* was detected only in the large intestine. *Mycoplasma* spp. was detected in 76% of large intestine samples, 68% of trachea samples, 63% of lung samples, 29% of spleen samples, and 5% of blood samples. *Plasmodium* spp. was found in 53% of lung and large intestine samples, 46% of trachea and blood samples, and 38% of spleen samples. In mammals, *C. jejuni* was detected in 100% of large intestine, trachea, and spleen samples and 50% of lung samples. *Mycoplasma* spp. was detected in 75% of large intestines, 70% of tracheae, 65% of lungs, 40% of spleens, and 45% of blood samples. *Plasmodium* spp. was only detected in the trachea with a high Ct value of 37.98.

### Histopathological analysis

Pathogens were detected in 82 (67.8%) of 121 animals examined. All patients did not show any clinical signs regarding these pathogens. Morphologically, it was difficult to confirm the effect of infection because most patients showed hemorrhage or tissue damage due to trauma. Twenty-two (22/82, 18.2%) cases, including 4 mammals (4/22, 18.2%) and 18 birds (18/22, 81.8%), showed histopathological lesions such as inflammation or necrosis in internal organs associated with infections. In mammals, only *Mycoplasma* spp. was detected from three raccoon dogs and one Korean water deer. Among them, in one raccoon dog, *Mycoplasma* spp. was detected in the lung, large intestine, and trachea. However, histopathological lesions related to pathogens were only found in the lungs (mild interstitial pneumonia) [15]. The other raccoon dog with *Mycoplasma* spp. detected in blood only showed hepatic necrosis. Thus, additional qPCR was performed and *Mycoplasma* spp. was detected in the liver. The last raccoon dog infected by endoparasites and ectoparasites showed inflammation in kidney, heart, pancreas, large intestine, stomach, lung, and spleen. However, *Mycoplasma* spp. was only detected in the large intestine, heart, pancreas, and kidney. In one Korean water deer rescued after a car accident, *Mycoplasma* spp. was detected in the lung and trachea. However, histopathological lesion regarding infection was not confirmed in the lung or trachea. Instead, chronic nephritis showed and additional qPCR detected *Mycoplasma* spp. in the kidney. In birds, *Mycoplasma* spp. were detected the most frequently (16/18, 88.9%), followed by *Plasmodium* spp. (5/18, 27.8%). *C. jejuni* (2/18, 11.1%), and *C. coli* (1/18, 5.6%). The association between detected pathogen and histopathological lesion was not distinct except for two cases (1 Chinese Little Bittern and 1 Eurasian Woodcock) of *Plasmodium* spp. detected in the liver (mild hepatitis) [16].

### **Molecular analysis of*Plasmodium*spp.**

**Fig. 1:**
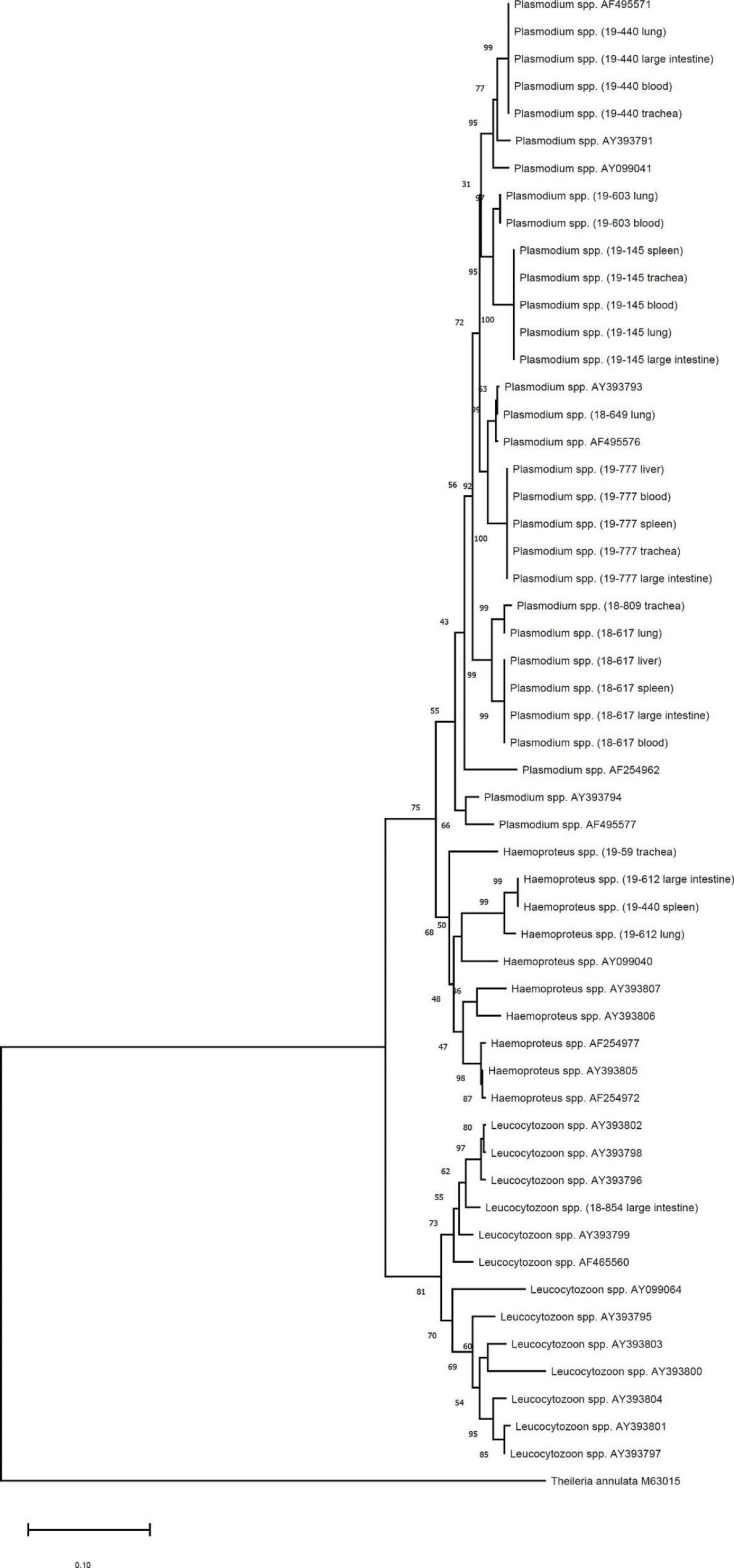
Sequence homologies of *Plasmodium* spp. detected in multiple organs in a single case. *Plasmodium* spp. detected in the same individual showed the same sequence except for two cases. Sequences obtained from this study are written in terms of the year of rescue and detected organs followed by species. Neighbor-joining phylogenetic analysis was performed with MEGA-X. Bootstrap percentage values are shown on branches

**Fig. 2:**
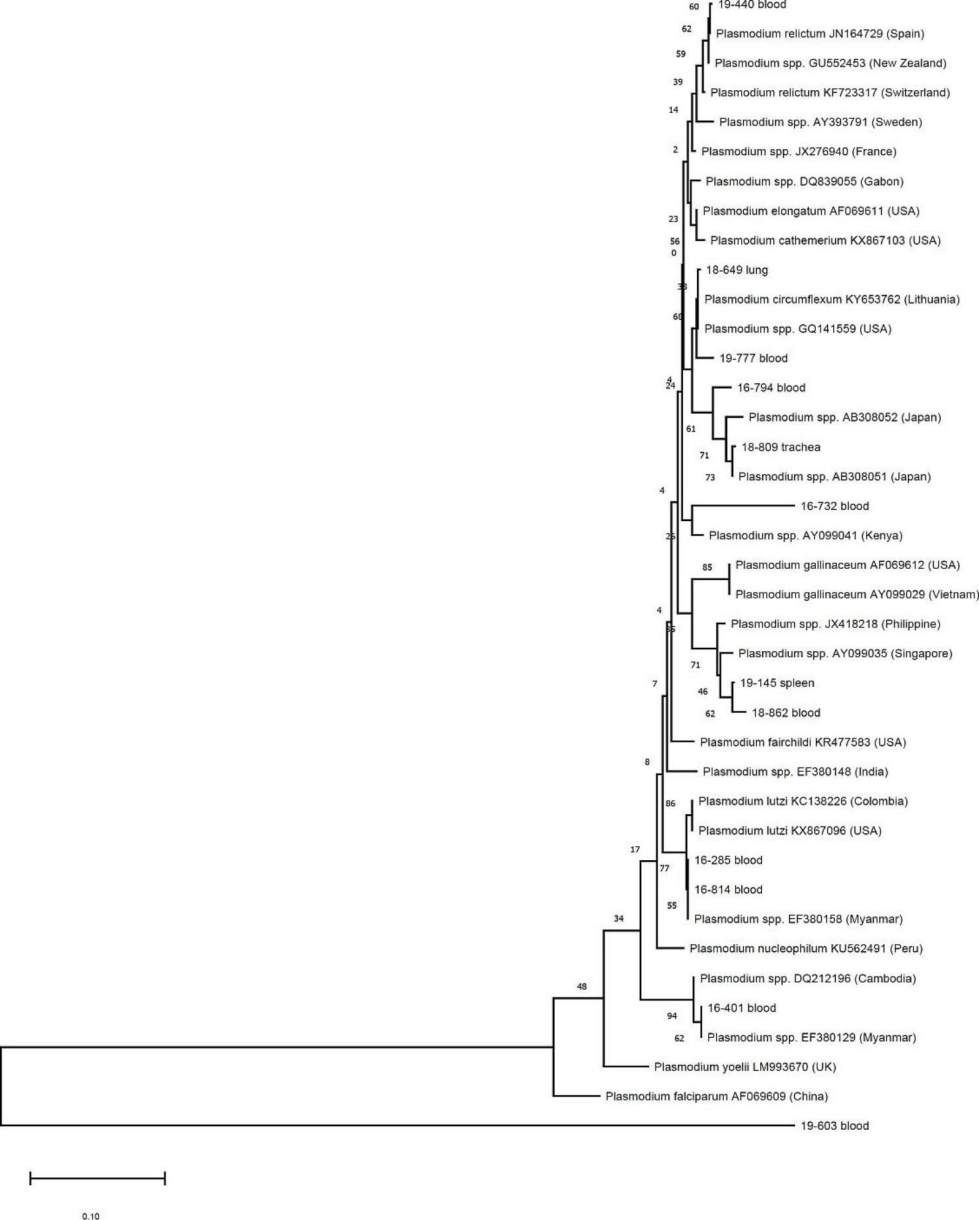
Phylogenetic analysis of *Plasmodium* identified in wild birds in this study, showing diverse genotypes. Sequences obtained from this study are written in terms of rescue year and detected organs. Neighbor-joining genetic analysis was performed using MEGA-X. Bootstrap percentage values are shown on branches

A phylogenetic analysis was conducted based on sequence homologies of *Plasmodium* spp. in each organ (Fig. 1). Among *Plasmodium* spp. positive samples through high-throughput qPCR platform and singleplex qPCR, two avian species of *Haemoproteus* spp. and one *Leucocytozoon* spp. were found in three animals through nested and conventional PCR. Therefore, these three animals were excluded. Through phylogenetic analysis, we confirmed that pathogens obtained from different organs of one animal showed homologous sequences except for two cases. For one animal, the sequence from the spleen showed *Haemoproteus* spp. from nested PCR. However, a conventional PCR revealed *Plasmodium* spp. For the other animal, sequences from the blood revealed *Plasmodium* spp. in nested PCR. However, in conventional PCR and sequencing, *Haemoproteus* spp. was detected. In the analysis of geographical distribution of genotypes (Fig. 2), samples found in a Korean water deer (*Hydropotes inermis*) (18–809) and a Chinese little bittern (*Ixobrychus sinensis)* (18–617) were most similar to the sequence of *Plasmodium* spp. found in Japan (AB308051, AB308052). The *Plasmodium* spp. detected in a Eurasian Woodcock (*Scolopax rusticola*) (19–777) and a spot-billed duck (*Anas poecilorhyncha*) (18–649) were the most similar to *Plasmodium circumflexum* (KY653762) found in Lithuania or *Plasmodium* spp. (GQ141559) found in the USA. The *Plasmodium* spp. detected in a Eurasian Jay (*Garrulus glandarius)* (19–440) was identical to *P. relicutum*, which was most similar to that found in Spain (JN164729). Samples found in a brown hawk-owls (*Ninox scutulata*) (19–145) showed sequences identical to the sequence of *Plasmodium* spp. found in Singapore (AY099035). The sample found in a cattle egret (*Bubulcus ibis*) (19–603) was not clustered with any sequences used in this study.

## Discussion

This study confirmed the presence of major infectious pathogens in the Republic of Korea in wildlife that died or euthanized at wildlife rescued center using a high-throughput platform. In this study, more than one pathogen was detected in 67.8% of examined wild animals. Pathogen detection was higher in wild birds (40/55, 72.7%) than in wild mammals (42/66, 63.6%). Clinical symptoms corresponding to detected pathogens were not observed in all animals during treatment periods. On the other hand, 18.2% of detected pathogens were confirmed to be associated with histopathological lesions regarding infections. Histopathological lesions related to detected pathogens were only shown in six patients (3 raccoon dogs, 1 Korean water deer, 1 Chinese Little Bittern, and 1 Eurasian Woodcock). Even in these six patients, specific lesions for each pathogen were not shown or no lesion was identified in any organs where pathogens were detected. Therefore, the detection of such pathogens was more likely to be from secondary infections or incidental finding due to the effect of trauma or other infections such as ectoparasites or endoparasites. Considering that all wild animals included in this study were rescued from anthropogenic accidents or injuries, or from serious ectoparasitic infections (severe scabies infection in raccoon dogs), wild animals are carrying pathogens as asymptomatic carriers.

Wild birds are considered as carriers of human camphylobacteriosis [17]. The transmission of this pathogen occurs through a fecal-oral route, causing waterborne outbreaks [18]. *Campylobacter* spp. is also a thermophilic pathogen. Birds are considered good reservoir of these pathogens because birds have higher body temperatures than mammals [19]. Patients carrying *Campylobacter* spp. showed common features consistent with those described in previous studies [17–19]. All patients were waterfowl or the rescued area was near water. In addition, more were detected in birds (14.5%) than in mammals (3%). In addition, patients did not show any symptoms corresponding to these pathogens such as enteritis. Since wild birds living near the water are more likely to asymptomatic reservoirs of *Campylobacter* spp., monitoring should be conducted for them.

In this study, *Mycoplasma* spp. had the highest prevalence (64.5%). However, clinical signs and histopathological lesions associated with these pathogens were rarely observed. Only four (4/78, 5.1%) patients showed histopathological lesions in organs from which pathogens were detected. Even in wild animals with histopathological lesions, the direct cause of death was trauma due to accidents, indicating that wild animals might be potential carriers of *Mycoplasma* spp. A variety of *Mycoplasma* spp. have been identified in wild animals worldwide. Although the pathogenesis has not been elucidated yet, *Mycoplasma* spp. is known to cause various diseases [20]. Further studies are necessary to identify the correlation between the presence of bacteria and pathological changes in wild animals.

In this study, in all wild animals in which *Plasmodium* spp. was detected in multiple organs, the same sequence was confirmed as a result of sequencing of pathogens detected for each organ except for two cases. Sequence comparison also revealed that *Plasmodium* spp. detected in the trachea of Korea water deer (*Hydropotes inermis*) (18–809) shared 99% sequence similarities with those detected in Chinese little bitterns (*Ixobrychus sinensis)* (18–617). It was unclear why *Plasmodium* spp., which is highly similar to that isolated from birds, was detected in organs of herbivores. However, cases of pathogenic induction by erythrocyte development of *Plasmodium* spp. have been reported [21]. Although rare, interspecies transmission cases indicate that mammals have contact with birds in the wild [22]. A more extensive study on the degree of *Plasmodium* spp. infection in wild mammals and its genetic similarity with avian isolates if *Plasmodium* spp. is needed in the future.

In a previous study conducted in the same area [23], *Plasmodium* spp. was detected in 6 (5.1%) out of 118 birds, whereas it was detected in 6 (10.9%) out of 55 birds in this study, showing a higher prevalence. *Plasmodium* spp. identified in the previous study were mainly similar to those identified in Asian countries regarding their genetic diversities, while *Plasmodium* spp. identified in this study became more diverse to include those detected in Europe or the United States. It can also be seen that genotypes between migratory birds or between migratory birds and resident birds are still similar. Sequences of a *Plasmodium* spp. isolated from a Korean water deer (*Hydropotes inermis*) (18–809) was most similar to that of a *Plasmodium* spp. isolated from Japan. They both were in the same clade as *Plasmodium* spp. detected from Chinese little bitterns (*Ixobrychus sinensis)* (18–617) and brown hawk owl (*Ninox scutulata*) (16–794). This may indicate that migratory birds’ role as mediators of *Plasmodium* spp. is at risk of expanding to mammals as well as resident birds. However, this study was limited to those with histopathological evaluations among those who died or were euthanized. Since screening criteria of samples were different from those of the previous study, direct comparison of prevalence was limited. A separate study should be conducted to analyze changes in the prevalence of *Plasmodum* spp. in the study area.

## Conclusion

Since pathogens of wild animals can also be pathogens of humans and livestock, various investigations of diseases centered on wild animal carcasses have been conducted. This study confirms that wild animals that appear healthy without having specific pathogen-specific lesions can carry pathogens, demonstrating that wild animals can be asymptomatic carriers of pathogens. Future studies on wildlife-borne diseases should use carcasses and determine the possibility of simple carriers simultaneously.

## Methods

### Sample Collection

A total of 121 wild animals (55 birds and 66 mammals) rescued between January 2018 and December 2019 that died or were euthanized during treatment were included in this study (Table 2). In the case of euthanizing wild animals because they could not be release into the wild, T-61 euthanasia solution (Embutramide, Mebenzonium iodide, Tetracaine hydrochloride infectable solution) was injected at 0.3 mg/kg under unconsciousness after deep inhalation anesthesia with isoflurane. In the course of treatment, animals had no specific clinical symptoms for infectious diseases. No other signs were observed in clinical laboratory tests (complete blood count and clinical chemistry analysis). After death or euthanasia, necropsy and histopathological examination were performed to determine the cause of death. During necropsy, four organ tissues (trachea, lung, large intestine [including stool], and spleen) were collected and stored in ethanol for screening of infectious agents. The peripheral blood collected and used for blood tests during medical treatment and stored was also included in the investigation of infectious agents.

**Table 2:** Information on wildlife species (scientific name) included in this study

Species	Common name (scientific name)	No. of patients
Bird	Green-backed heron (*Butorides striata*)	1
	Magpie (*Pica pica*)	2
	Chinese oriole (*Oriolus chinensis*)	1
	Chinese little bittern (*Ixobrychus sinensis*)	1
	Woodcock (*Scolopax rusticola*)	1
	Oriental turtle dove (*Streptopelia orientalis*)	1
	Humming bird (*Trochilidae*)	1
	Sparrow hawk (*Accipiter nisus*)	2
	Scops owl (*Otus scops*)	3
	Brown hawk-owl (*Ninox scutulata*)	8
	Eagle owl (*Bubo bubo*)	6
	Eurasian Jay (*Garrulus glandarius*)	1
	Gy heron (*Ardea cinerea*)	5
	Large egret (*Egretta alba modesta*)	3
	Intermediate egret (*Mesophoyx intermedia*)	1
	Domestic pigeon (*Columba livia domestica*)	1
	Goshawk (*Accipiter gentilis schvedow*)	3
	Cattle egret (*Bubulcus ibis*)	7
	Kestrel (*Falco tinnunculus interstinctus*)	5
	Hoopoe (*Upupa epops saturata*)	1
	Spot-billed duck (*Anas poecilorhyncha*)	1
Mammal	Korean water deer (*Hydropotes inermis*)	37
	Raccoon dog (*Nyctereutes procyonoides*)	25
	Western roe deer (*Capreolus pygargus*)	1
	Marten (*Martes flavigula koreana*)	1
	Wild boar (*Sus scrofa*)	1
	Leopard cat (*Prionailurus bengalensis*)	1

### Nucleic acid extraction and reverse transcription

Tissues were washed with distilled water to remove ethanol. Blood and tissue samples were ground separately using TissueLyser II (Qiagen, Hilden, Germany) with nuclease-free water and then mixed together. The total volume was 200 or 250 µL. DNA and RNA were extracted simultaneously using a MagMAX™ Total Nucleic Acid Isolation Kit (Thermo Fisher Scientific, Waltham, MA, USA) according to the manufacturer’s instructions. Nucleic acid extraction was also conducted for positive samples using a QIAamp DNA Mini Kit (Qiagen). For RNA viruses, reverse transcriptase PCR was performed using a high-capacity cDNA reverse transcription kit (Thermo Fisher Scientific). Extracted nucleic acids were stored at -20℃.

### High-throughput qPCR platform

A high-throughput qPCR platform panel was designed to simultaneously detect 19 pathogens (*Brucella* spp., *Campylobacter jejuni*, *Campylobacter coli*, *Chlamydia pneumoniae*, *Chlamydia psittaci, Chlamydia abortus*, *Mycoplasma haemofelis*, *Mycoplasma haemominutum*, *Mycobacterium avium, Mycobacterium bovis*, *Mycoplasma* spp., Japanese encephalitis virus, Severe fever with thrombocytopenia syndrome virus, West Nile virus, Newcastle virus, avian influenza, *Cryptococcus neoformans*, *Leucocytozoon* spp, and *Plasmodium* spp.) in duplicates (48 samples could be examined simultaneously). PCR amplification was performed in a total volume of 5 µL. The reaction mixture contained 1.2 µL nucleic acid, 2.5 µL TaqMan^®^ Fast Advanced Master Mix (Thermo Fisher Scientific), and 1.3 µL of nuclease-free water. The reaction mixture was then loaded onto a plate using an automated sample loading system (Thermo Fisher Scientific). In the plate, each through-hole contained 33 nL reaction mixture. The primer and probe sets were obtained from published sources [24–41]. Additional information about primers and probes can be found in additional file [see Additional file 1]. The PCR protocol consisted of 40 cycles of 95 °C for 15 s and 60 °C for 1 min.

### Singleplex qPCR

Singleplex qPCR was performed for high-throughput qPCR platform-positive samples to determine which tissues or blood samples contained the pathogen. It was laso performed for additional organ tissues showing histopathologic lesions. For nucleic acid amplification, 2 µL of nucleic acid from each organ and blood was used as a template in a final volume of 20 µL with TaqMan^®^ Fast Advanced Master Mix (Thermo Fisher Scientific), 0.2 µM of each primer, and 0.1 µM of each probe. The thermal cycler protocol consisted of incubation at 50 °C for 2 min, 95 °C for 20 s, and 40 cycles at 95 °C for 1 s and at 60 °C for 20 s. The primer and probe were the same as those designed for the high-throughput qPCR platform panel. After qPCR, all positive samples were sequenced and compared to BLAST database to determine pathogenic organisms.

### **Molecular Analysis for*Plasmodium*spp.**

Two gel-based PCRs and sequencing was performed for phylogenetic analysis of identified *Plasmodium* spp. One was nested PCR for comparing sequence homologies of identified hemoparasites in a single case and the other one was a conventional PCR for comparing geographical distribution of genotypes. Both steps of nested PCR were performed in a final volume of 50 µL using a HotStarTaq Master Mix Kit (Qiagen). The template consisted of 2 µL of nucleic acid isolated from each organ and blood. The mixture was denatured for 15 min at 95^o^C, followed by 40 cycles at 95^o^C for 45 s, 50^o^C for 45 s, and 72^o^C for 1 min, and finally extended at 72^o^C for 7 min before maintaining at 4^o^C. The first PCR product was used as a template for the second PCR. For nested PCR, HameNF1-HaemNR3 primers were used for the first PCR and HaemF-HaemR2 primers were used for the second PCR. PCR amplicons had an expected size of 617 bp for nested PCR [42]. For conventional PCR, the first reaction was performed using 3760 F-4292Rw2 primers. If the fragment was not amplified, a follow-up reaction was performed with smaller fragments using F1-4292Rw2, F3-4292Rw2, and 3760 F-R1 primer pairs. PCR amplicons had an expected size of 400–500 bp [43, 44].

Phylogenetic analysis was conducted by comparing base pair of *cytochrome b* sequence. Phylogenetic analysis was performed for two purposes: (1) to compare sequence homology of *Plasmodium* spp. detected in each organ (Fig. 1); and (2) to estimate geographical distribution of *Plasmodium* spp. mainly transmitted by wild birds (Fig. 2). For the phylogenetic tree for confirming sequence homology, a sequence of *Theileria annulata* (GenBank accession number: M63015) was used as outgroup. For comparing geographical diversity, the phylogenetic tree was made using a sequence of *Plasmodium* spp. (GenBank accession numbers AY099029 and JN164729) as outgroup. Neighbor-joining genetic analysis was performed using MEGA-X. Numbers on branches indicate bootstrap values based on 1,000 replicates.

### Electronic supplementary material

Below is the link to the electronic supplementary material.

Supplementary Material 1

## Data Availability

This article contains all data created or analyzed throughout the investigation.

## Abbreviations

EID: Emerging infectious disease
AI: Avian influenza
qPCR: Quantitative polymerase chain reaction
PCR: Polymerase chain reaction
*C.jejuni*: *Campylobacter jejuni*
*C.coli*: *Campylobacter coli\*
*P. relicutum*: *Plasmodium relicutum*
