# Supplementary material for: Surveillance of wild animals carrying infectious agents based on high-throughput screening platform in the Republic of Korea
Source: BMC Vet Res. 2023 Sep 14;19:158. doi: 10.1186/s12917-023-03714-0 (PMC10500733; doi:10.1186/s12917-023-03714-0)
Supplement: Supplementary file 1 — Supplementary Material 1 [file 12917_2023_3714_MOESM1_ESM.pdf]

Primer and probe for high-throughput qPCR and singleplex qPCR

	Agent	Target gene	Primer or probe (5′/3′ labels)	Sequence (5′ to 3′)
Bacteria	*Brucella* spp.	IS*711*	F	GCTTGAAGCTTGCGGACAGT
			R	GGCCTACCGCTGCGAAT
			Probe (FAM/QSY)	AAGCCAACACCCGGCCATTATGGT
	Campylobacter jejuni	*MapA*	CJ_F	CTGGTGGTTTTGAAGCAAAGATT
			CJ_R	CAATACCAGTGTCTAAAGTGCGTTTAT
			CJ_P (FAM/QSY)	TTGAATTCCAACATCGCTAATGTATAAAAGCCCTTT
	Camphylobacter coli	*Gyrase subunit A*	CC_F	AAGCTCTTATTGTTCTAACCAATTCTAACA
			CC_R	TCATCCACAGCATTGATTCCTAA
			CC_P (FAM/MGB)	TTGGACCTCAATCTCGCTTTGGAATCATT
	Chlamydia pneumoniae	*ompA*	CP Fw	AAGGGCTATAAAGGCGTGCT
			CP Rev	TGGTCGCAGACTTTGTTCCA
			CP Probe (FAM/QSY)	TCCCCTTGCCAACAGACGCTGG
	Chlamydia psittaci	*ompA*	CppsOMP1-F	CACTATGTGGGAAGGTGCTTCA
			CppsOMP1-R	CTGCGCGGATGCTAATGG
			CppsOMP1-S (FAM/MGB)	CGCTACTTGGTGTGAC
	Chlamydia abortus	*ompA*	CpaOMP1-F	GCAACTGAGACTAAGTCGGCTACA
			CpaOMP1-R	ACAAGCATGTTCAATCGATAAGAGA
			CpaOMP1-S (FAM/QSY)	TAAATACCACGAATGGCAAGTTGGTTTAGCG
	Mycoplasma hemofelis	16s rRNA	Hfelis_F	GGGGCCAAGTCAAGTCATC
			Hfelis_R	GCGAATTGCAGCCTTTTATC

		Hfelis_P (FAM/QSY)	TYAAGAACACCAGAGGCGAAGGCG
Mycoplasma haemominutum	16s rRNA	Hmin_F	GGGGCCAAGTCAAGTCATC
		Hmin_R	GCGAATTGCAGCCTTTTATC
		Hmin_P (FAM/QSY)	TACCATTGTAGCACGTTYGCAGCCC
Mycobacterium avium	IS*901*	IS901-se	GTGATCAAGCACCTTCGGAA
		IS901-pe	GCTGCGAGTTGCTTGATGAG
		IS901TM (FAM/QSY)	AACAACATCGACACGATCGCCGACAA
Mycobacterium bovis	*mpt64*	mtb-F	GTGAACTGAGCAAGCAGACCG
		mtb-R	GTTCTGATAATTCACCGGGTCC
		mtb-P (FAM/QSY)	CCGGCATTCGGCGCTATCGATA
Mycoplasma spp	16s rRNA	Myco16sQF1	GCAAAGCTATAGAGATATAGTAGAGGT
		Myco16sQR	GTTGCGYTCGTTGCRGGAC
		Myco16sQProbe (FAM/MGB)	TGGTGCATGGTTGTC
		Myco16sQF2	GCRAAGCTATAGARATATAGTGGAGGT
		Myco16sQR	GTTGCGYTCGTTGCRGGAC
		Myco16sQProbe (FAM/MGB)	TGGTGCATGGTTGTC
		Myco16sQF3	GCAATGCTATAGAGATATAGCGGAGGT
		Myco16sQR	GTTGCGYTCGTTGCRGGAC
		Myco16sQProbe (FAM/MGB)	TGGTGCATGGTTGTC
		Myco16sQF4	GCAAAGCTATGGAGACATAGTGGAGGT
		Myco16sQR	GTTGCGYTCGTTGCRGGAC

			Myco16sQProbe (FAM/MGB)	TGGTGCATGGTTGTC
			Myco16sQF5	GCAAAGTTATGGAAACATAATGGAGGT
			Myco16sQR	GTTGCGYTCGTTGCRGGAC
			Myco16sQProbe (FAM/MGB)	TGGTGCATGGTTGTC
**Virus**	Japanese encephalitis virus	*Core*	JE-multi-F	AGAACGGAAGAYAACCATGACTAAA
			JE-multi-R	CCGCGTTTCAGCATATTGAT
			Multi-probe (FAM/MGB)	ACCAGGAGGGCCCGG
	Severe fevere with thrombocytopenia syndrome virus	L segment	L-F-3	AGTCTAGGTCATCTGATCCGTTYAG
			L-R-3	TGTAAGTTCGCCCTTTGTCCAT
			L-Probe-3 (HEX/QSY)	AATGACAGACGCCTTCCATGGTAATAGGG
	Westnile virus	NS2A	WNRT-F	CGGAAGTYGRGTAKACGGTGCTG
			WNRT-Re	CGGTWYTGAGGGCTTACRTGG
			WNV (FAM/MGB)	WCCCCAGGWGGACTG
	Newcastle virus	Matrix genes	F	TGACACCATCACCGACCAC
			R	CTCCCTCTCTGCCCATTTCTT
			P (FAM/QSY)	TTTTAACGCTCCGCAGGCAC
	Avian influenza	H5 HA	H5-HA FP	CTTGCGACTGGGCTCAGAAAT
			H5-HA RP	TTTGGGTGGATTCTTTGTCTGC
			H5-HA P (FAM/MGB)	CATTCCCTGCCATCC
		H7 HA	H7-HA FP	ATTGGACACGAGACGCAATG
			H7-HA RP	TTCTGAGTCCGCAAGATCTATTG

			H7-HA P (FAM/QSY)	TAATGCTGAGCTGTTGGTGGCA
		NPsegment	NP FP	ACCAGAAGATKTGTCMTTCCAGGG
			NP RP	TACTCCTCCGCATTGTCTCCGAAG
			NP P (FAM/MGB)	AAGGCAACGAACCC
		M gene	M-Flu1	CTTCTAACCGAGGTCGAAACGTA
			M-Flu2	GGATTGGTCTTGTCTTTAGCCA
			M-Fluprob (FAM/MGB)	CTCGGCTTTGAGGGGGCCTGA
**Fungus**	*Crpytococcus neoformans*	ITS2-rDNA	CneoFwd	GCCGCGACCTGCAAAG
			CneoRev	GGTAATCACCTTCCCACTAACACAT
			CneoProbe (FAM/MGB)	ACGTCGGCTCGCC
**Pariasites**	*Leucocytozoon* spp.	cyt-b	Leuc_01F	CTGCTTTCATGGGTTATGTCTTACCA
			Leuc_01R	AAGTGTAATACAAAGAATCTTTTTAATGTT
			Leuc_01P (FAM/MGB)	AATCCACCACAAACCC
			Leuc_02F	GTTACTTACCTTTATCATGGAGTAGTGGTT
			Leuc_02R	CTCATTTGACCCCATGGTAAGACAT
			Leuc_02P (FAM/MGB)	CCCATGAAAGCAGTTACAATA
			Leuc_03F	ATTAATGATCCAACATTAAAAAGATTCTTT
			Leuc_03R	AGGATTAGTCGTACCTTGAATATGTAAGAA
			Leuc_03P (FAM/MGB)	TTCCCATTCGTAGCTTTAG
	Plasmodium spp.	18S rDNA	F	AGCCTGAGAAATAGCTACCACATCTA
			R	TGTTATTTCTTGTCACTACCTCTCTTCTTT

	P (FAM/QSY)	CAGCAGGCGCGTAAATTACCCAATTC
